# The Effects of Violent Video Game Characters' Race on Implicit and Explicit Racial Attitudes

**DOI:** 10.1002/ijop.70157

**Published:** 2025-12-28

**Authors:** Tailson Evangelista Mariano, Victoria da Costa Perman, Carlos Eduardo Pimentel, Isabella Leandra Silva Santos, Cícero Roberto Pereira

**Affiliations:** ^1^ Catholic University of Pernambuco Recife Brazil; ^2^ Federal University of Paraíba João Pessoa Brazil; ^3^ University of Lisbon Lisbon Portugal

**Keywords:** implicit attitudes, ingroup identification, racial bias, stereotype activation, video games

## Abstract

This study investigates how racialized representations in violent video games influence implicit and explicit racial attitudes, and whether these effects are moderated by the player's racial identity. Grounded in the General Aggression Model and Evaluative Conditioning theory, 140 participants were randomly assigned to view a violent gameplay video featuring either a Black or a White character. Implicit attitudes were assessed using the Implicit Association Test, and explicit attitudes were measured with a biological racism scale. Results revealed a significant main effect of character race on implicit attitudes: participants exposed to the Black character showed stronger pro‐White/anti‐Black biases. Moderation analysis indicated that this effect was significant among White participants but not among Black participants. Conversely, Black participants exhibited a significant reduction in explicit racism after exposure to the violent Black character, whereas White participants showed no change. Although moderation effects were marginally significant, the crossover pattern suggests that ingroup identification and stereotype activation may operate in opposite directions depending on viewer identity. These findings advance understanding of media‐induced racial bias and highlight the need for more inclusive character representations in interactive media. Implications for theory, game design, and media literacy are discussed.

## 1 Introduction

The use of video games has grown exponentially in recent decades, becoming one of the most prevalent forms of entertainment worldwide (Duarte 2025). However, the literature has increasingly pointed to potential negative effects of this form of media consumption on individuals' attitudes and behaviour (Anderson and Bushman 2002; Greitemeyer and Mügge 2014). The General Aggression Model (GAM) posits that exposure to violent media primes aggression‐related knowledge structures, increases arousal, and fosters hostile affect, ultimately facilitating aggressive behaviour (Bushman and Anderson 2020). Beyond aggression, the GAM has also been applied to explain how repeated exposure to media stereotypes might influence social judgements, including racial attitudes (Anderson and Bushman 2002).

Empirical studies have shown that video games, particularly violent ones, tend to depict Black characters more frequently in stereotypical roles, such as criminals or gang members (Dill et al. 2005; Williams et al. 2009; Yang et al. 2014). These portrayals may reinforce pre‐existing cultural stereotypes that associate Blackness with danger and deviance. Such exposure to racially stereotyped violent content may activate implicit associations between social categories (e.g., race) and evaluative content (e.g., threat), consistent with the mechanisms described in the theory of Evaluative Conditioning (EC; Walther et al. 2011). According to this framework, repeated pairings between a stimulus (e.g., a Black character) and a negative outcome (e.g., violence) may shape attitudes automatically, even without the individual's conscious awareness.

Despite the growing body of literature on the influence of media on racial attitudes, most studies have examined the effects of passive media (e.g., news, television), with fewer investigations focusing on interactive formats such as video games (Behm‐Morawitz and Ortiz 2013). Moreover, few studies have explored how these effects differ according to participants' social group membership. According to the Multidimensional Model of Racial Identity (MMRI; Sellers et al. 1998), race may be central to the self‐concept and influence how racial information is interpreted. Thus, individuals may react differently to stereotyped portrayals of racial groups depending on their own racial identification.

Research has shown that stereotype activation tends to occur more strongly when the content reinforces outgroup stereotypes (Devine 1989; Eberhardt et al. 2004; Yang et al. 2014). In contrast, ingroup members may experience stereotype threat or reactance when exposed to negative portrayals of their group, sometimes resulting in an increase in group identification or resistance to bias (Ramos et al. 2020; Sanders et al. 2024). Therefore, the race of the observer may moderate the effects of character race on racial attitudes.

Racial attitudes can be conceptualised along both explicit and implicit dimensions. Explicit attitudes refer to evaluations that are deliberate and consciously accessible, typically measured via self‐report instruments. In contrast, implicit attitudes are automatic, often unconscious, and assessed through indirect measures such as the Implicit Association Test (IAT; Greenwald et al. 2022). The IAT measures the strength of automatic associations between concepts (e.g., race) and evaluations (e.g., good/bad) by analysing response latency patterns. Both forms of attitudes can be affected by media exposure, though they may follow different cognitive pathways (Greenwald et al. 2022; Pimentel et al. 2022).

The current study examines whether the race of a violent video game character affects players' implicit and explicit racial attitudes. Previous research has shown that avatar race can influence stereotype activation and intergroup bias in gaming contexts (Cicchirillo 2015; Yang et al. 2014), suggesting that racial cues embedded in violent media may shape evaluative associations and social perceptions. Building upon this literature, participants in the present study were randomly assigned to watch a video of a violent video game featuring a Black or White avatar. Implicit attitudes were measured using the IAT, whereas explicit beliefs were assessed through a biological racism scale (Vala et al. 2012).

Based on the GAM and EC theories, we hypothesized that exposure to a violent Black character would increase pro‐White/anti‐Black implicit bias, particularly among White participants (H1). Conversely, drawing on the MMRI, we expected that Black participants would exhibit lower levels of explicit racism after exposure to a violent Black character, potentially due to ingroup identification and resistance to internalised stereotypes (H2). These hypotheses aim to clarify how violent, racialised media content may differentially affect automatic and controlled components of racial attitudes.

## 2 Method

### 2.1 Design and Participants

To test these hypotheses, we employed a between‐subjects experimental design to investigate the effects of character race in violent video games on implicit and explicit racial attitudes. A total of 140 participants aged 18 to 62 years (*M* = 25, SD = 7.84) were recruited via social media. Although the age range was broad, most participants were young adults, and age showed no significant correlation with either implicit or explicit attitude scores (*r* < 0.10, *p* > 0.05), suggesting that developmental variability did not confound the findings. Participants self‐identified as White (*n* = 87) or Black (*n* = 53), with the majority being female (67.9%). Sample size was determined a priori using G*Power software to ensure 80% statistical power for detecting small to medium effects (Cohen's *d* = 0.30; Verma and Verma 2020).

Participants were randomly assigned to one of two experimental conditions: Exposure to a violent video game with a White character; or exposure to a violent video game with a Black character. Inclusion criteria required participants to be over the age of 18 and have minimal prior gaming experience. This decision was intended to reduce familiarity effects with violent video game content, ensuring that participants' responses reflected spontaneous cognitive activation to the racial manipulation rather than previously learned gameplay schemas.

### 2.2 Procedures

The study was conducted entirely online via the Qualtrics platform to ensure a secure and controlled environment for data collection. Participants were automatically randomised into the experimental conditions using Qualtrics' built‐in randomizer function, which ensured an equal allocation across groups (i.e., Black vs. White video game character). Randomisation checks confirmed that the two groups did not differ significantly in age, gender, or prior gaming experience (all ps > 0.05), indicating adequate group balance. This procedure strengthened internal validity by reducing potential selection biases while maintaining the anonymity and automation typical of online experiments.

Exposure consisted of watching a 1‐min video from the game GTA V. The videos were carefully designed to highlight the character's race and violent behaviour. Each video showed the character engaging in identical violent actions, including physical aggression, weapon use and interactions with non‐playable characters (NPCs) in hostile scenarios. In addition, visually salient cues such as skin colour, facial features and culturally relevant clothing were highlighted, with close‐ups enhancing the salience of the manipulated variable. This combination of violent actions and racial representation ensured that the manipulation effectively reflected both the aggression and racial dimensions relevant to the study's hypotheses (Hollingdale and Greitemeyer 2013; Heng et al. 2017). The exposure duration was limited to 1 min to function primarily as a cognitive activation (priming) stimulus rather than a prolonged behavioural task. This brief exposure is theoretically aligned with the proximal processes described by the GAM (Bushman and Anderson 2020), which posits that even short‐term violent media exposure can activate aggression‐related and stereotype‐consistent cognitions. Furthermore, concise and intense video segments are sufficient to elicit measurable cognitive effects while maintaining participant attention and minimising fatigue in online settings.

After watching the assigned video, participants completed the IAT. Responses were automatically recorded in the Qualtrics platform and response times were used to calculate the *D*‐score. Participants then completed the Biological Racism Scale to assess explicit racial attitudes. Demographic data such as age, gender, race, and previous gaming experience were also collected via the platform. At the end of the session, participants received a debriefing in which they were informed of the true purpose of the study. By using Qualtrics, data security, anonymity, and confidentiality were ensured, and ethical standards were maintained throughout the research process.

### 2.3 Video Game Manipulation

Grand Theft Auto V (GTA V), a video game known for its graphic violence (Teng et al. 2011), was used for the study. Two 1‐min videos were created that emphasised the character's racial identity (Black vs. White) in an identical violent context. The clips were edited to ensure equivalence in duration, actions, and visual composition, differing only in the race of the central character. Both versions depicted the avatar performing the same aggressive behaviours (e.g., physical assaults and weapon use) within identical environmental settings and camera angles. Visual cues such as skin colour, facial features, and culturally relevant clothing were highlighted to ensure the salience of the racial manipulation. To confirm that the manipulation was perceived as intended, a manipulation check was conducted. Participants rated the character's perceived race immediately after the exposure. Results indicated a significant difference between conditions, *t*(138) = −2.49, *p* = 0.014, *d* = −0.42, confirming that participants accurately identified the race of the avatar in each condition. This procedure followed previous research investigating a race‐based manipulation of violent conditions (Hollingdale and Greitemeyer 2013; Yang et al. 2014).

## 3 Measures

### 3.1 Implicit Racism Measure

We used the Qualtrics‐based IAT (Do Bú et al. 2024) to assess automatic associations between racial categories (Black and White) and evaluative concepts (good and bad). Faster responses to stereotype‐consistent pairings (‘White/Good’ and ‘Black/Bad’) compared to stereotype‐inconsistent pairings (‘White/Bad’ and ‘Black/Good’) indicate greater anti‐Black implicit bias. The *D*‐score was calculated following Greenwald et al. (2022), using the average response latency and pooled standard deviation.

### 3.2 Explicit Racism Measure

The Biological Racism Scale (Vala et al. 2012) was used to assess explicit beliefs about the biological inferiority of racial groups. The scale consists of 10 items rated on a Likert scale from 1 (*strongly disagree*) to 7 (*strongly agree*). Sample items include: ‘Certain racial groups are biologically more intelligent than others’. The internal consistency of the scale was satisfactory (*α* = 0.83).

### 3.3 Socio‐Demographic Questions

Demographic data, including age, gender, race, and prior gaming experience, were collected to characterise the sample.

## 4 Data Analysis

The data were analysed using the JASP software (version 0.19.2; JASP Team 2024) and the jamovi software (version 2.6; The jamovi project 2024). Descriptive analyses were first conducted to characterise the sample and assess data distribution. A 2 (Character Racial Category: Black vs. White) × 2 (Participant Racial Group: Black vs. White) between‐participants Analysis of Variance (ANOVA) was used to examine the effects on implicit and explicit racial attitudes.

To assess moderation effects, we tested for a significant interaction between the racial category of character and the participant's racial group. Simple effects analyses were conducted to determine whether the manipulated racial category of character influenced implicit and explicit racial bias within each racial group (White or Black participants). Internal consistency of the instrument was assessed using Cronbach's alpha.

## 5 Results

**FIGURE 1:**
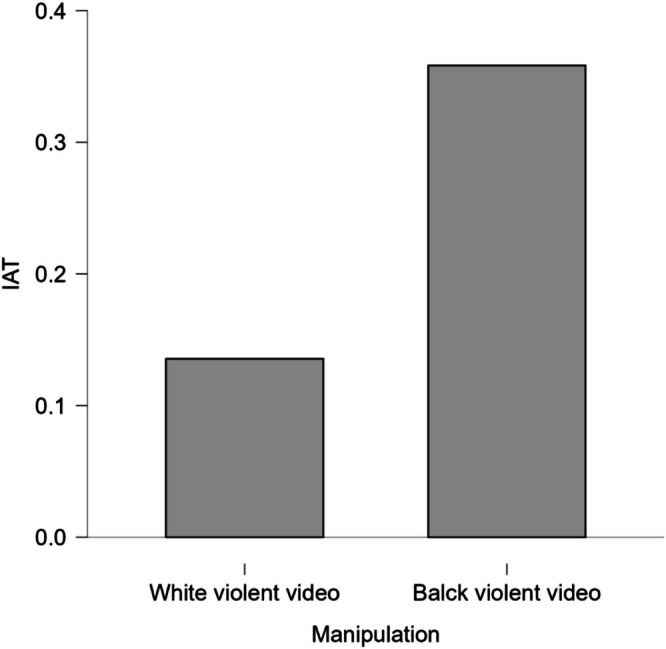
Mean scores on the Implicit Association Test (IAT) by character race condition (Black vs. White). Higher scores indicate stronger implicit pro‐White/anti‐Black bias.

To examine the effects of character race on racial attitudes, both analysis of variance (ANOVA) and moderation analyses were conducted. An initial ANOVA tested the main effect of character race on implicit attitudes, as measured by the IAT. The results revealed a statistically significant effect, *F*(1, 138) = 6.23, *p* = 0.014, partial *η* ^2^ = 0.043, 95% CI [0.002, 0.127]. Participants exposed to a violent Black character showed higher levels of implicit racial bias (*M* = 0.36, SD = 0.45) compared to those exposed to a violent White character (*M* = 0.14, SD = 0.60). This finding supports the hypothesis that racialised representations in violent video games heighten automatic negative associations. Figure 1 displays this effect.

In contrast, the ANOVA for explicit racial attitudes, measured using the Biological Racism Scale, did not reach statistical significance, *F*(1, 138) = 0.87, *p* = 0.352, partial *η* ^2^ = 0.006. Average scores did not differ meaningfully between participants who viewed the Black character (*M* = 30.46, SD = 5.18) and those who viewed the White character (*M* = 31.29, SD = 5.31), indicating that the manipulation influenced only implicit attitudes. This dissociation aligns with theoretical models suggesting that implicit and explicit attitudes operate through distinct psychological processes.

To examine whether the effect of character race varied by participant race, a moderation analysis using linear regression was conducted. The interaction between character race (Manipulation) and participant race (Race2) was marginally significant for implicit attitudes, *B* = −0.305, SE = 0.179, *Z* = −1.70, *p* = 0.089. Simple slope analyses showed that among White participants (−1 SD), exposure to a violent Black character significantly increased implicit racial bias (*B* = 0.364, SE = 0.124, *Z* = 2.94, *p* = 0.003), suggesting heightened stereotype activation. Among Black participants (+1 SD), the effect of the manipulation was not significant (*B* = 0.068, SE = 0.124, *Z* = 0.55, *p* = 0.582), indicating no meaningful shift in implicit attitudes. This pattern supports the hypothesis that White participants are more susceptible to stereotype activation when exposed to violent, racialised content.

For explicit attitudes, the interaction between manipulation and participant race was also marginally significant, *B* = −3.78, SE = 1.967, *Z* = −1.92, *p* = 0.054. Simple slope analysis indicated that among Black participants (+1 SD), exposure to a violent Black character led to a significant reduction in explicit racism scores (*B* = −3.022, SE = 1.36, *Z* = −2.22, *p* = 0.026), possibly reflecting an ingroup identification effect. Among White participants (−1 SD), the manipulation had no significant impact on explicit attitudes (*B* = 0.645, SE = 1.365, *Z* = 0.47, *p* = 0.636). This cross‐pattern of results highlights a nuanced dynamic in which implicit and explicit attitudes respond differently to violent media depending on the viewer's racial identity.

## 6 Discussion

The findings of this study provide empirical support for the central hypotheses and deepen our understanding of the psychological mechanisms through which racialised media content shapes social attitudes. Specifically, exposure to violent Black characters significantly increased implicit racial bias, particularly among White participants, while explicit racial attitudes remained largely unchanged, except among Black participants, who showed a notable reduction in biological racism. These patterns align with the GAM (Bushman and Anderson 2020), which suggests that violent media stimuli heighten the accessibility of aggressive and stereotype‐consistent cognitions. For White participants, such exposure likely activated long‐standing associative links between race and violence, reinforcing implicit biases. In contrast, Black participants may have processed the same content through the lens of ingroup identification, which can function as a psychological buffer against internalising negative stereotypes. This divergence underscores the importance of considering the intersection between media exposure and viewers' social identities, and reflects how implicit and explicit processes operate through distinct but interacting cognitive systems.

Additionally, EC theory complements this interpretation by explaining how repeated associations between race and violence can reinforce negative attitudes in the long term (Walther et al. 2011). Together, these theories provide a nuanced understanding of the mechanisms underpinning media influence on racial attitudes, emphasising the dynamic interplay between immediate cognitive activation and long‐term associative learning.

The dissociation found between implicit and explicit attitudes reinforces existing literature on self‐reported prejudice measures. Explicit attitudes are often shaped by social norms and conscious self‐regulation (Devine 1989; Todd et al. 2011). While participants may have consciously adjusted their explicit responses to avoid appearing prejudiced, the implicit attitudes captured by the IAT reveal automatic negative associations. This pattern is consistent with the concept of aversive racism, where individuals outwardly reject prejudice but continue to exhibit subtle, automatic biases in ambiguous or stress‐inducing situations (Do Bú et al. 2024). Such biases can be particularly pronounced when stereotypes are activated by media exposure, as seen in this study where violent Black characters heightened implicit biases among White participants. These findings emphasise the need to examine how repeated exposure to stereotypical representations contributes to the persistence of automatic prejudices, even in individuals who consciously endorse egalitarian values.

Critically, the moderation analyses offer additional nuance to our understanding of how media exposure interacts with individual characteristics. Based on Hayes's (Igartua and Hayes 2021) model of conditional process analysis, the present study found moderation effects that approached, but did not reach, conventional levels of statistical significance. These results should therefore be interpreted with caution. Nonetheless, their direction and consistency with the theoretical framework provide preliminary evidence that participant race may condition the relationship between character race and racial attitudes. Specifically, simple slope analyses showed that White participants exhibited stronger implicit racial bias after exposure to violent Black characters, whereas this effect was not observed among Black participants.

Conversely, for explicit attitudes, Black participants showed a reduction in racist beliefs, while no change was observed among White participants. Overall, while the crossover pattern observed should be interpreted with caution given the marginal levels of significance, it remains theoretically meaningful in light of prior evidence on stereotype activation and ingroup identification (Sellers et al. 1998; Yang et al. 2014). Rather than drawing firm causal conclusions, these results are best viewed as preliminary indications of how racial identity may shape the cognitive processing of violent, racialized media content.

The moderation effect on implicit attitudes is consistent with the literature on stereotype activation, which shows that individuals from dominant groups are more likely to exhibit increased bias when exposed to stereotypical portrayals (Yang et al. 2014; Eberhardt et al. 2004). For White participants, the exposure to violent Black avatars may have activated culturally learned associations between Blackness and criminality, thus increasing automatic negative evaluations. In contrast, for Black participants, the absence of this effect suggests that shared group membership with the avatar may act as a cognitive buffer, interrupting the internalisation of stereotypical content. This supports the MMRI (Sellers et al. 1998), which posits that ingroup affirmation can serve a protective function in identity‐relevant contexts.

The decrease in explicit racism among Black participants exposed to violent Black characters is particularly noteworthy. Rather than reinforcing internalised stereotypes, the content may have triggered identity salience or reactance, leading participants to reject essentialist or biologically based racist beliefs (Ramos et al. 2020; Sanders et al. 2024). This possibility aligns with literature on stereotype threat and identity mobilisation, in which stigmatising content can, under certain conditions, prompt members of marginalised groups to reaffirm their group in positive ways. The absence of any significant change in explicit attitudes among White participants further reflects the limits of explicit measures in capturing more automatic, less regulated reactions to media content.

The results further underscore the importance of employing complementary strategies to measure attitudes in social psychology. While explicit measures, such as self‐report scales, provide insights into conscious and regulated responses, implicit measures like IAT reveal biases that operate at an automatic level, beyond conscious awareness (Pimentel et al. 2022). This dual‐approach methodology ensures a more comprehensive understanding of how media exposure influences both overt and covert forms of prejudice. By integrating these measures, the study captures the nuanced interplay between controlled and automatic processes in racial attitudes, contributing to the broader literature on the mechanisms underpinning media effects.

From a theoretical perspective, this study advances the understanding of how interactive media shapes racial attitudes by integrating GAM and EC. While GAM provides a framework for understanding the activation of aggressive cognitions, EC details the associative mechanisms that may perpetuate biases (Walther et al. 2011; Hollingdale and Greitemeyer 2013). This combined theoretical approach underscores the importance of investigating both immediate and long‐term effects of media exposure, offering a comprehensive model for interpreting the cognitive and social implications of such representations. Examining this theoretical interplay further could illuminate how certain media features amplify or mitigate these effects over time (McGovern and Otten 2024).

The findings have important implications for the design of interactive media, public policy, and media literacy education. Video game developers should critically assess the portrayal of racial characters in their games. Incorporating diverse and non‐stereotypical representations can mitigate the reinforcement of negative implicit biases. For instance, creating complex Black characters in non‐violent roles may counteract existing stereotypes and promote more equitable social perceptions (Dill and Burgess 2012). Additionally, game developers could benefit economically from responding to consumer demand for more inclusive media, as public awareness of representation issues continues to grow (Shor and van de Rijt 2024).

Educational media literacy initiatives should aim to enhance consumers' awareness of the potential subconscious effects of media exposure. Programs that foster critical thinking about media content can help individuals become more mindful of implicit messages and develop resistance to biased portrayals (Ramasubramanian 2015). Interactive workshops and tools tailored to younger audiences may be particularly effective in fostering these critical skills.

Furthermore, public policies could be directed towards regulating content that perpetuates harmful racial stereotypes. Regulatory bodies might consider guidelines that encourage or incentivise the production of media content with balanced and fair representations of all racial groups. Such policies could contribute to reducing societal biases and promoting inclusivity. These strategies are especially relevant in contexts where racism often operates at implicit levels, such as Brazil (Camino et al. 2001; Sanders et al. 2024).

Despite using a well‐established paradigm, the present study extends previous work in several important ways. First, it simultaneously examined both implicit and explicit dimensions of racial attitudes within the same experimental framework, providing a more comprehensive understanding of how different evaluative systems respond to racialized media content. Second, by incorporating participants' racial identity as a moderator, the study advances prior findings (e.g., Cicchirillo 2015; Yang et al. 2014) that largely focused on majority‐group participants. This intersectional approach highlights how race‐specific cognitive processes, such as stereotype activation among dominant groups and ingroup affirmation among marginalised groups, may operate in opposite directions. Finally, the study contributes new evidence from a racially diverse, non‐U.S. sample, expanding the cross‐cultural scope of research on media effects and implicit bias, an area still underrepresented in the literature.

While the results provide evidence of the impact of character race on racial attitudes, several limitations should be acknowledged. First, the study relied on short video clips instead of active gameplay. Although this approach allowed for tighter experimental control and reduced participant burden, it may have limited immersion and ecological validity. Future studies should investigate whether active interaction with racially stereotyped characters produces stronger or more persistent effects. Second, the manipulation was designed to ensure equivalence across scenes but was not pretested before data collection. Although the manipulation check confirmed that participants accurately perceived the racial identity of the characters, future research should include systematic pretesting of stimuli to further enhance internal validity.

Third, data were collected online through a convenience sample, which may not fully represent the broader population of video game players. Studies using stratified or community‐based samples could examine whether these effects generalise across different demographic and cultural groups. Finally, although the inclusion of participants with minimal prior gaming experience ensured unbiased reactions to the stimuli, future investigations could also examine habitual players to evaluate how familiarity with violent or racialised content moderates attitudinal responses. Longitudinal and cross‐cultural designs would also be valuable to assess whether the effects observed here persist over time and across distinct media environments.

In conclusion, this study contributes to the growing body of literature on media effects by elucidating the complex interplay between violent video game content, racial representations, and implicit attitudes. The integration of GAM and EC theories offers a robust framework for understanding these dynamics, with significant implications for media production, consumption, and regulation. Addressing the nuanced ways in which media shapes social cognition is essential for fostering a more equitable and inclusive society.

## Author Contributions

All authors contributed substantially to this work and approved the final version of the manuscript for publication. Specifically: Tailson Evangelista Mariano contributed to the conception and design of the study, the collection, analysis, and interpretation of data, and the drafting and critical revision of the manuscript for important intellectual content. Victoria da Costa Perman contributed to the collection, analysis, and interpretation of data, as well as the drafting and critical revision of the manuscript for important intellectual content. Carlos Eduardo Pimentel contributed to the study's conception and design, data analysis and interpretation, and manuscript drafting and critical revision for important intellectual content. Isabella Leandra Silva Santos contributed to the analysis and interpretation of data, drafting and critical revision of the manuscript for important intellectual content. Cícero Roberto Pereira contributed to the study's conception and design, data analysis and interpretation, and manuscript drafting and critical revision for important intellectual content.

## Funding

This work was supported by the Fundação de Amparo à Ciência e Tecnologia do Estado de Pernambuco (FACEPE) through the Institutional Scientific Initiation Scholarship Program (PIBIC), awarded to Victoria da Costa Perman under grant number BIC‐1225‐7.07/24.

## Ethics Statement

All procedures performed in studies involving human participants were in accordance with the ethical standards of the institutional research committee at the Universidade Católica de Pernambuco (CAAE: 87660725.5.0000.5206) and with the 1964 Helsinki Declaration and its later amendments or comparable ethical standards.

## Consent

Informed consent was obtained from all individual adult participants included in the study.

## Conflicts of Interest

The authors declare no conflicts of interest.

## Data Availability

The data that support the findings of this study are available from the corresponding author upon reasonable request.
